# Design of Dual-Mode Local Oscillators Using CMOS Technology for Motion Detection Sensors

**DOI:** 10.3390/s18041057

**Published:** 2018-04-01

**Authors:** Keum-Won Ha, Jeong-Yun Lee, Jeong-Geun Kim, Donghyun Baek

**Affiliations:** 1 Microwave Embedded Circuit & System (MECAS) Lab., School of Electrical Engineering, Chung-Ang University, 84 Heukseok-ro, Dongjack-gu, Seoul 06974, Korea; justlikefeel@nate.com (K.-W.H.); lostria1985@gmail.com or lostria@cau.ac.kr (J.-Y.L.); 2 Integrated Radar Lab., Department of Electronic Engineering, KwangWoon University, 20 Gwangun-ro, Nowon-gu, Seoul 01897, Korea; junggun@gmail.com or junggun@kw.ac.kr

**Keywords:** CMOS, dual-mode, local oscillator (LO), FMCW radar, motion detection, sensor

## Abstract

Recently, studies have been actively carried out to implement motion detecting sensors by applying radar techniques. Doppler radar or frequency-modulated continuous wave (FMCW) radar are mainly used, but each type has drawbacks. In Doppler radar, no signal is detected when the movement is stopped. Also, FMCW radar cannot function when the detection object is near the sensor. Therefore, by implementing a single continuous wave (CW) radar for operating in dual-mode, the disadvantages in each mode can be compensated for. In this paper, a dual mode local oscillator (LO) is proposed that makes a CW radar operate as a Doppler or FMCW radar. To make the dual-mode LO, a method that controls the division ratio of the phase locked loop (PLL) is used. To support both radar mode easily, the proposed LO is implemented by adding a frequency sweep generator (FSG) block to a fractional-N PLL. The operation mode of the LO is determined by according to whether this block is operating or not. Since most radar sensors are used in conjunction with microcontroller units (MCUs), the proposed architecture is capable of dual-mode operation by changing only the input control code. In addition, all components such as VCO, LDO, and loop filter are integrated into the chip, so complexity and interface issues can be solved when implementing radar sensors. Thus, the proposed dual-mode LO is suitable as a radar sensor.

## 1. Introduction

**Figure 1:**
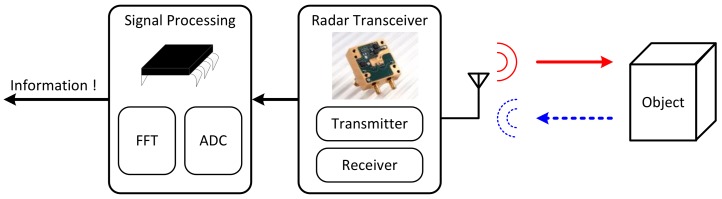
Conceptual diagram explaining the general operation of the radar sensor.

A radar sensor is divided into signal processor and radar transceiver as shown in Figure 1. The signal generated by the transmitter is radiated by the antenna, and the signal reflected from the target is detected by the receiver. To obtain information about the object, an appropriate algorithm must be applied to the received signal at the signal processor.

In the early days of its development, radar was mainly used for military purposes such as detecting missiles and fighter aircraft. Military radars need to detect objects located hundreds of kilometers away. For this reason, a very large antenna is used to amplify the transmitted signal and increase the sensitivity of the received signal. Recently, radar technology has been applied for other purposes such as air traffic control systems and weather observation systems, by using characteristics of radar that can detect objects at long distance. Compound semiconductor devices are still used in these applications. Even though these devices are expensive and their power consumption is large, the durability and performance of the device itself are excellent. Most RF transceivers used in these radar systems are implemented by connecting components of compound semiconductor devices on the printed circuit board (PCB) [1,2,3]. These radars are not suitable for sensors to detect targets within tens of meters, such as unmanned security sensors, motion detection sensors and automotive driving assistant systems (ADAS), because of their size, cost, and power dissipation. With the development of the complementary metal-oxide-semiconductor (CMOS) process and integrated chip (IC) technology, it is possible to design a radar transceiver in X-band, K-band, and W-band with low power and low cost. Therefore, many studies on implementing CMOS radar transceiver [4,5,6,7,8,9,10,11,12] have been actively conducted, recently. In addition, studies on signal processing algorithms that improve the performance of radar sensors have also been carried out [13,14,15,16,17,18,19].

Radar sensors can be arranged in two categories, depending on the type of radiated signal. One is pulsed-radar using intermittent signals [6,7,20] and the other is continuous wave (CW) radar. The most significant difference between these two structures is the type of information obtained. Time of flight (ToF) refers to the time taken for the signal transmitted from the radar to return after it has been reflected by the target. The operation of the pulsed-radar involves directly detecting this time. In contrast, the shifted frequency occurring at the moment of reflection by the target of the radiated signal due to ToF is information detected by the CW radar [21,22]. 

**Figure 2:**
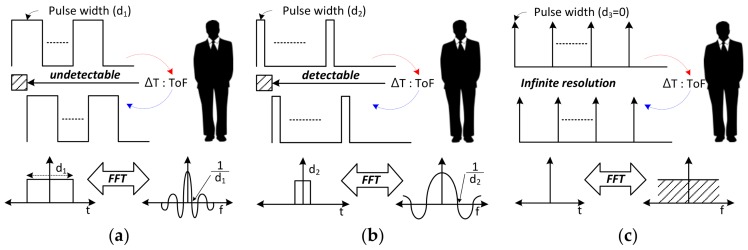
Relationship between frequency bandwidth and range resolution. (**a**) In the case where the pulse width of the transmitted signal has the largest pulse width; (**b**) In the case where the pulse width of the transmitted signal is narrower than that in the previous condition; (**c**) As the most ideal condition, the transmitted signal is a pulse-train.

Figure 2 shows the operation of the pulsed-radar under the same ToF with different pulse width. As shown in Figure 2a, the transmitted signal has the largest pulse width (*d*_1_). In this case, the target could not be detected because of the range ambiguity problem. This problem occurs when the transmitted and received signals overlap. Figure 2b shows the operation of pulsed-radar when a much narrower pulse width (*d*_2_) is used compared to Figure 2a. In this case, the target can be detected because the range ambiguity problem does not occur. An ideal case using a delta pulse train with a pulse width of zero is shown in Figure 2c. Comparing the results of the three cases, the pulsed radar detects target more accurately as the pulse width narrows. As shown in the bottom of Figure 2, a wider frequency bandwidth is required to generate narrower pulse. Therefore, the pulsed-radar has an advantage for using in radar sensors which are allocated with a wide frequency bandwidth. Radar sensors that detect motion detection are mainly implemented in the X-band. Since the maximum frequency bandwidth assigned to the X-band is 50 MHz [23], a CW radar is used rather than pulsed-radar.

**Figure 3:**
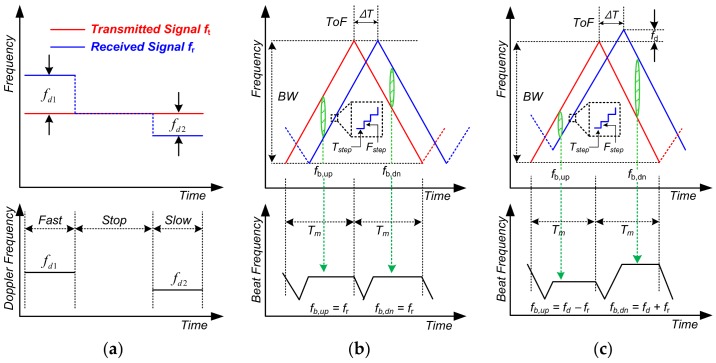
Plots of the operation method of CW radar (**a**) Doppler mode operation. As an example of FMCW mode operation, triangular waveform is described when the target is (**b**) stationary and (**c**) moving condition.

There are two ways to operate the CW radar. One is a Doppler mode using a fixed frequency, and the other is a FMCW mode using a periodically swept frequency within a certain range. The operation method of CW radar is described in Figure 3. In Doppler mode, the CW radar detects the frequency difference that occurs in proportion to the moving speed of the object. As shown in the figure, the faster the target moves, the larger the frequency difference that appears (Figure 3a). Although detecting the momentary movement of the target is an advantage, no signal is detected when the movement is stopped. This is a problem when the CW radar operates in the Doppler mode.

In FMCW mode, the up-chirp and down-chirp intervals are periodically repeated. During the up-chirp interval, the frequency becomes faster and reaches the maximum frequency. On the contrary, during the down-chirp interval, the frequency becomes slower and returns to the starting frequency. The time taken to perform the frequency chirp once is called the chirp time, and the frequency variation range is the chirp bandwidth. As shown in Figure 3, the difference between the Doppler and FMCW mode is that information can be obtained even if the target is stationary or moving, because the received signal is delayed by ToF compared to the transmitted signal (Figure 3b,c). The frequency difference between the transmitted and received signal at the same time is called the beat frequency. The process for obtaining the beat frequency in ideal condition that the shape of chirp waveform is perfectly linear will be explained. As shown in Figure 3b, since the Doppler frequency is not generated at the stationary target, only the distance information is obtained. The equations of the transmitted and received signal are expressed as follows:

(1) $$T_{x}\left(t\right)=\{\begin{array}{c} \frac{B}{T_{m}}\times t\;\left(0\leq t<T_{m}\right)\\ -\frac{B}{T_{m}}\times \left(t-T_{m}\right)+B\;\left(T_{m}\leq t<2T_{m}\right) \end{array}$$

(2) $$R_{x}\left(t\right)=\{\begin{array}{c} \frac{B}{T_{m}}\times \left(t-∆T\right)\;\left(0\leq t<T_{m}\right)\\ -\frac{B}{T_{m}}\times \left(t-T_{m}-∆T\right)+B\;\left(T_{m}\leq t<2T_{m}\right) \end{array}$$

where *B* is the chirp bandwidth, *t* is time, *T_m_* is the chirp time, ∆*T* is the ToF.

From the difference between Equations (1) and (2), the beat frequency can be obtained as follows:

(3) $$f_{b,up}=f_{b,dn}=\frac{B}{T_{m}}\times ∆T=f_{r}$$

The ToF is determined by distance between FMCW radar and target, so that only the distance information is expressed by the beat frequency, *f_r_*. As described Figure 3c, since the Doppler frequency is occurred when the target moves at a specific speed, the equation of received signal is changed as follows:

(4) $$R_{x}\left(t\right)=\{\begin{array}{c} \frac{B}{T_{m}}\times \left(t-∆T\right)+f_{d}\;\left(0\leq t<T_{m}\right)\\ -\frac{B}{T_{m}}\times \left(t-T_{m}-∆T\right)+B+f_{d}\;\left(T_{m}\leq t<2T_{m}\right) \end{array}$$

where *f_d_* is Doppler frequency that occurs when the target moves at a specific speed.

From the difference between Equations (1) and (4), the beat frequency can be written as:

(5) $$f_{b,up}=\frac{B}{T_{m}}\times ∆T-f_{d}=f_{r}-f_{d}\;\left(0\leq t<T_{m}\right)$$

(6) $$f_{b,dn}=\frac{B}{T_{m}}\times ∆T+f_{d}\;=f_{r}+f_{d}\left(T_{m}\leq t<2T_{m}\right)$$

Based on these equations, the range information is calculated by adding Equation (5) and (6):

(7) $$f_{r}=\frac{f_{b,up}+f_{b,dn}}{2}$$

The speed information is calculated by subtracting Equation (5) from Equation (6):

(8) $$f_{d}=\frac{f_{b,dn}-f_{b,up}}{2}$$

As can be seen in Equations (7) and (8), both the distance and velocity information can be obtained in the FMCW mode. However, it is difficult for the FMCW radar to generate a perfect linear chirp waveform like the ideal condition, so the chirp waveform is generated by accumulating *F_step_* every *T_step_* to make a waveform similar to the ideal condition, as shown in the Figure 3. Since microwave is moving at the speed of light in the air, the ToF of the object near the sensor is very short. If ToF is smaller than *T_step_*, beat frequency is not generated and information about target can’t be obtained. Thus, the function of FMCW mode might not be exerted when the detection object is near the sensor. Therefore, if the CW radar can be operated in the dual mode, the disadvantage which occurs when the CW radar operates in only one mode can be overcome.

**Figure 4:**
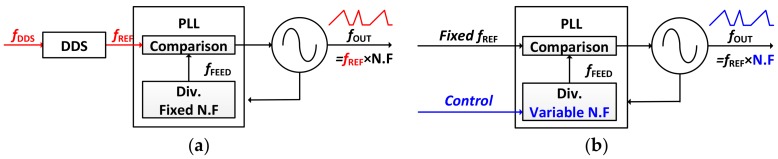
General structure of a dual-mode LO using PLL. (**a**) Reference clock modulation method; (**b**) Division ratio control method.

The operating mode of the CW radar depends on the local oscillator (LO), which is responsible for generating the emitted signal of the radar transceiver. As described in Figure 4, a dual-mode LO can be implemented in two different ways using a phase locked loop (PLL). One way is reference clock modulation method [24,25,26] and the other is a division ratio control method [27,28,29,30]. Because of using a direct digital synthesizer (DDS), the former has an advantage in that the output frequency could be changed very fast with precise resolution, but its disadvantages make it unsuitable for miniaturized radar sensors. To operate the DDS, an additional clock is required that is faster than the reference clock of the PLL. In addition, a look-up table is needed to reconstruct the sine wave, so a storage device such as a ROM is required. Finally, a higher order low-pass filter (LPF) is needed to remove the harmonics of the output frequency.

**Figure 5:**
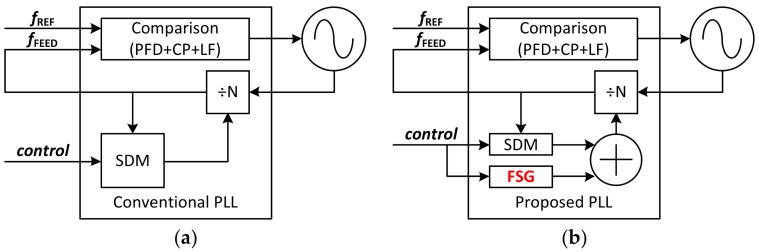
Comparison of dual-mode LO (**a**) the conventional and (**b**) the proposed structure using division ratio control method.

Figure 5 shows the difference between the conventional (Figure 5a) and the proposed (Figure 5b) architecture of the dual mode LO using division ratio control method. To operate dual-mode in the conventional structure, the division ratio of the PLL must be directly controlled by external software. However, in the proposed structure, the division ratio of the PLL is controlled by frequency sweep generator (FSG) block. As an input to the FSG block, information about the chirp bandwidth and time is stored in this block. When the proposed LO operates in FMCW mode, the division ratio of the PLL is automatically changed by the FSG block based on the stored information. Otherwise, the proposed LO operates in Doppler mode because the division ratio is fixed. Thus, the operation mode is determined by whether the FSG block is operating or not.

Doppler or FMCW mode operation, independently or simultaneously, is usually adopted for radar sensors. To implement both modes in one radar module, there are many drawbacks such as the interface complexity, the amount of power consumption, and the size of module. These disadvantages can reduce the competitiveness of radar sensor. In this paper, an architecture for realizing the dual-mode LO to support the both radar mode very easily. The proposed structure is implemented by adding a frequency sweep generator (FSG) block to a fractional-N PLL. When operating in FMCW mode, only the chirp time and bandwidth are determined, and the desired modulation waveform can be generated by this LO. If the FSG block is turned off, the proposed LO is changed to Doppler mode. The advantage of a radar sensor applying the proposed structure is that the function of the sensor can be improved according to how to use. For example, if an object is detected in Doppler mode and the position is tracked in FMCW mode, the function of the unmanned security system or the automatic lighting control sensor can be further improved. Thus, the proposed structure is suitable for motion detection sensors.

The rest of this paper is organized as follows: Section 2 presents more precise explanation of proposed structure with simulation waveform and detailed block diagrams. Comparison table with previous studies and measurement data are shown in Section 3. Finally, conclusions are presented in Section 4.

## 2. Proposed Dual-Mode Local Oscillator Design

**Figure 6:**
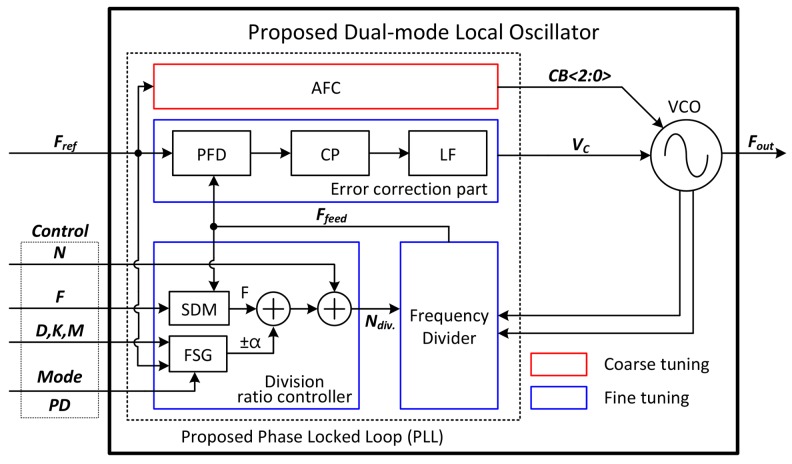
Block diagram of the proposed dual-mode LO.

Figure 6 shows a block diagram of the proposed dual-mode LO. As shown in this figure, it is divided into the voltage-controlled oscillator (VCO) and the proposed phase locked loop (PLL). The following is a brief description of the sub-blocks that make up the PLL. The automatic frequency calibrator (AFC) determines the capacitor bank code of the VCO. The feedback signal (*F_feed_*) is generated by dividing the VCO frequency at the frequency divider (FD) block. The error correction part is composed of a phase frequency detector (PFD), charge pump (CP) and loop filter (LF). The phase and frequency difference between the reference clock (*F_ref_*) and the *F_feed_* are detected by PFD and CP. These errors are converted to DC voltage by the LF. The division ratio controller (DRC) is the most important block in the proposed LO, consisting of a sigma-delta modulator (SDM) and a frequency sweep generator (FSG). Because the division ratio of the PLL is controlled by the DRC, dual-mode operation can be easily realized by adjusting the control code.

The inputs of the proposed structure are the reference clock and control codes. Among the control codes, the power down (PD) determines the operating mode of the LO. If the frequency sweep generator (FSG) block is turned-off, the LO operates in Doppler mode, otherwise in FMCW mode. The ‘*Mode*’ determines whether the LO starts from up-chirp or down-chirp when the modulation waveform is generated in FMCW mode. Finally, *N*, *F*, *D*, *K* and *M* determine the division ratio of the proposed PLL. In Doppler mode, the division ratio of the PLL consists of *N*, representing the integer part, and F is responsible for the fractional part. Alpha is the output of the FSG, which is only needed when operating in FMCW mode. Alpha represents a value added or subtracted to the division ratio to make the output frequency swept within a certain range. Since the output frequency is expressed as a function of the input values, the following equations give the output frequency of LO in each mode:

(9) $$f_{out\_Doppler\_mode\;}=F_{ref}\times \left(N.F\right)$$

(10) $$f_{out\_FMCW\_mode}=F_{ref}\times \left(N.F\pm \alpha \right)$$

The parameters of the proposed dual-mode LO are summarized in Table 1. A 38.4 MHz crystal oscillator is used as the reference clock of the proposed PLL. Considering the nonlinearity [5,30,31] and the accuracy problem [32,33,34,35] that might occur in the radar sensor, the loop bandwidth of the PLL is 500 kHz and the target in-band phase noise performance is about −80 dBc/Hz. In FMCW mode, the chirp time can be varied from 0.1 to 1 ms, and the maximum modulation bandwidth is 750 MHz.

### 2.1. Voltage Controlled Oscillator (VCO)

**Figure 7:**
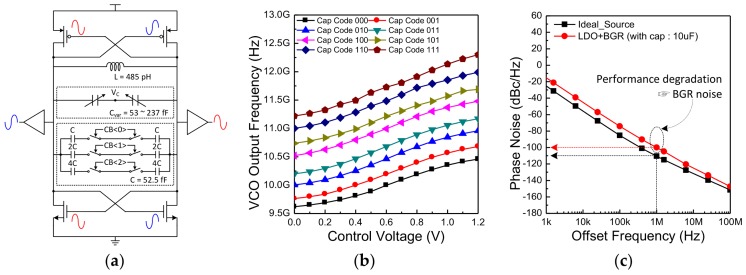
(**a**) A detailed schematic of the VCO used in the proposed structure; (**b**) The output frequency range is described as a function of the capacitor bank and gate voltage of the varactor; (**c**) The phase noise performance of the VCO.

Various studies have been conducted on low-power VCOs. Among them, class-C VCO and current-reuse VCO are representative structures [36,37,38,39,40]. However, there are several drawbacks in applying these structures to radar transceiver. The class-C VCO requires an additional circuit to determine the optimum bias point. In addition, the problem of the current-reuse VCO is the phase and amplitude mismatch of the generated differential signal. Thus, to implement a radar transceiver without these problems, complementary cross-coupled VCO is used in the proposed architecture. A detailed schematic of the VCO is described (Figure 7a). The VCO consists of a P-type metal oxide semiconductor (PMOS) and N-type metal oxide semiconductor (NMOS) as a cross-coupled pair, an inductor, a capacitor bank for coarse tuning, and a varactor for fine tuning. As shown in the figure, depending on the input 3-bit signal (*CB<2:0>*) the switches in the capacitor bank are turned on or off. In other words, one of the eight curves is selected by this value (Figure 7b). Fine tuning means adjusting the gate voltage of the varactor so that the frequency is varied along the selected curve. The designed output frequency range of the VCO is 9.7–12.4 GHz. There is an overlap interval between each capacitor code to generate all frequencies without error when AFC operates. The phase noise simulation results are depicted in Figure 7c. When using an ideal voltage source, the phase noise performance is −110 dBc/Hz at 1 MHz offset. The actual implemented VCO is designed to be powered by a low dropout regulator (LDR) and bandgap reference (BGR). Although the phase noise performance of the VCO is degraded by about 10 dBc/Hz due to the noise of the BGR, there is no problem in meeting the target specification.

### 2.2. Proposed Phase Locked Loop (PLL)

#### 2.2.1. Automatic Frequency Calibrator (AFC)

The AFC operates before the fine-tuning operation. The AFC receives *F_ref_*, *F_feed_*, and counting value (*M*) as input, determines the VCO capacitor back code, and generates a signal that initiates the fine tuning operating at the end of the AFC operation. In general, for N-bit AFC, this operation is repeated N − 1 times to determine the final output value. The output of the AFC starts at the center value, 100 code, since 3-bit AFC operates as a binary search algorithm. The operating principle of the AFC is to compare the results by accumulating several cycles of *F_ref_* and *F_feed_* signals.

**Figure 8:**
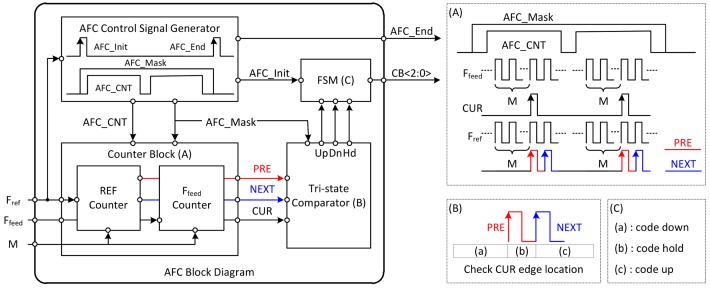
Entire block diagram of the AFC.

An entire block diagram of the AFC and a brief description of its operation are described in Figure 8. The AFC consists of an AFC control signal generator, counter block, tri-state comparator, and finite state machine. The AFC control signal generator makes four signals that determines the operation timing of the other blocks. AFC_Init indicates the moment when the operation of the AFC starts; AFC_End indicates that the operation of the AFC is finished and is used as a trigger for the fine tuning operation. The AFC_MASK is generated by using these two signals. The operation of the AFC is effective only when the voltage level of the AFC_MASK signal remains high. AFC_CNT is a signal that determines the time at which the counter block operates.

As shown in section (A) on the right side of Figure 8, the role of the counter block is to generate three signals (*PRE*, *NEXT*, *CUR*) by accumulating the period of *F_ref_* and *F_feed_*. The down-counting method is used in the REF counter and *F_feed_* counter, where the counting value is subtracted by 1 from input value (*M*) at the rising edge of two signals (*F_ref_, F_feed_*) which are entered to counter block. When the voltage level of AFC_CNT is changed from supply voltage to ground, the counting value is set to the initial value (*M*) again. This moment indicates that accumulating operation of the two signals (*F_ref_* and *F_feed_*) signal has been completed once. Each time this operation is completed, the counter block generates *PRE*, *NEXT*, and *CUR* pulses. During the time that the AFC_CNT signal remains low, the tri-state comparator performs a comparison using the three output signals of the counter block to decide whether to keep the AFC output value changed or not. As described in section (B) and (C) on the right side of Figure 8, unless the rising edge of CUR pulse is located in region (b), the output code is changed to up or down by the finite-state machine (FSM).

**Figure 9:**
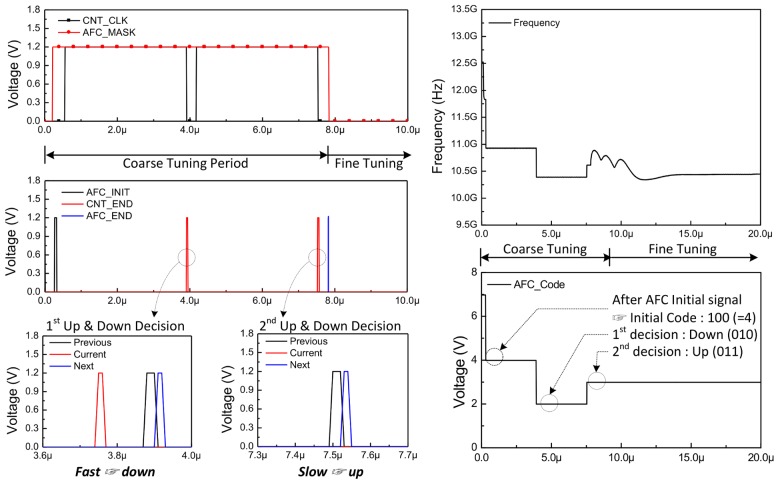
The simulation result of the AFC.

The simulation result of the AFC is depicted in Figure 9. The graph on the left shows the simulation waveforms of AFC internal signals. The graph on the right shows an example in which the entire LO operates, including the AFC operation, and the frequency is fixed at 10.5 GHz. As shown in this figure, it is confirmed that the interval maintaining high voltage in the AFC_CNT signal appears twice due to 3-bit AFC. The figure shows that the comparison operation is performed at the end of the accumulation operation. In the first comparison operation, because the *CUR* pulse is generated before the *PRE* and *NEXT* pulses, the accumulated value of the *F_feed_* is smaller than that of *F_ref_*. In this case, the tri-state comparator decides to lower the capacitor bank code from 100 code to 010 code. In the second comparison case, the tri-state comparator determines to increase the capacitor bank code from 010 to 011 code because the *CUR* pulse is not generated.

#### 2.2.2. Error Correction Part

**Figure 10:**
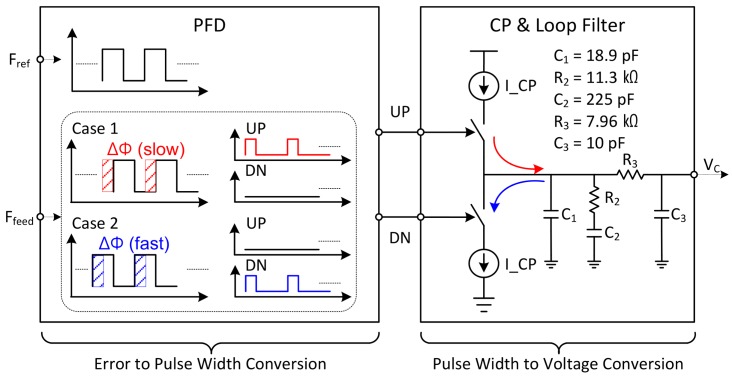
The conceptual block diagram on how to operate the error correction part.

The error correction part consists of a phase frequency detector (PFD), a charge pump (CP), and a loop filter (LF). The conceptual block diagram on how to operate the error correction part is described in Figure 10. The basic operation principle is as follows. The phase and frequency of the *F_feed_* signal is compared to the *F_ref_* in the PFD. The PFD converts the error between these two signals into a pulse. By using the two pulses generated from the PFD, the CP controls the amount of current flow into the LF. Finally, the LF accumulates the incoming current and converts it into a DC voltage. The voltage of the LF is equal to the gate voltage of the varactor in the VCO, so that the frequency of the VCO is finely controlled. For example, when the *F_feed_* signal is slower than *F_ref_* signal (case 1), the up-pulse is generated from PFD, so that the voltage of LF goes up. If the *F_feed_* signal is faster, the voltage of LF goes down (case 2). Finally, if there is no difference between the phase and the frequency, the output frequency of the LO (*F_out_*) is fixed. Under this condition, the voltage of LF is not changed, because the up and down switches of the CP are turned on for the same duration. Since LF accumulates the error within several times, it determines the reaction speed of the PLL. This reaction rate is called the loop bandwidth of the PLL. Generally, the loop bandwidth of the PLL depends on the CP current and the size of capacitor used in the LF. In the proposed PLL, the CP current value is 50 μA, and a third-order low-pass filter is used. The loop bandwidth of the PLL is set to 500 kHz. Each component value of the LF is shown in the figure.

#### 2.2.3. Frequency Divider (FD)

**Figure 11:**
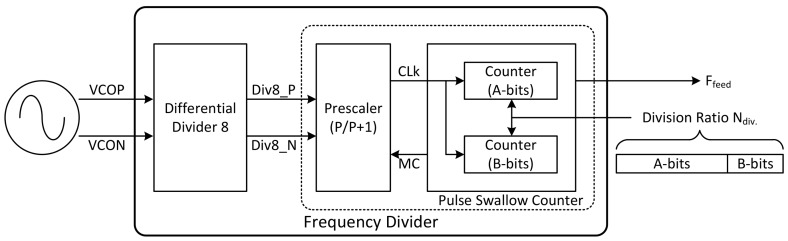
The overall block diagram of the frequency divider.

The overall block diagram of the frequency divider (FD) is depicted in Figure 11. The function of this block is to divide the VCO frequency according to the division ratio. This block is composed of the differential divider and the pulse swallow counter.

**Figure 12:**
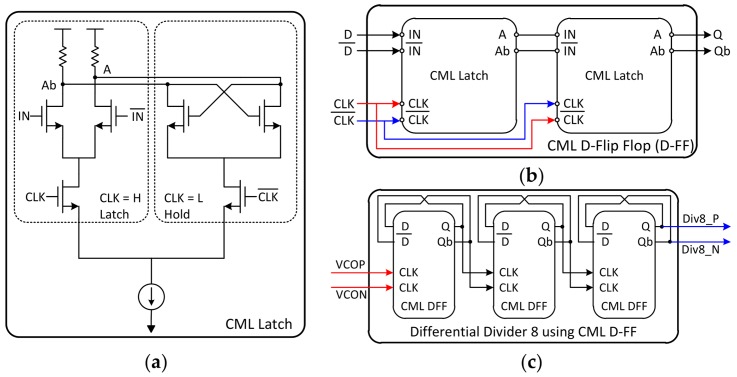
The detailed schematic of the differential divider, expressed up to the basic device level. The schematic of the CML latch is shown in (**a**); and the CML D-FF is described in (**b**); The schematic of the differential divider is illustrated in (**c**).

The function of the differential divider is to divide the VCO frequency by eight, because the output frequency of the VCO is too high to be used as the clock of the pulse swallow counter. In this case, current-mode logic (CML) is used to implement the differential divider. Figure 11 shows the detailed schematic of the differential divider expressed up to the basic device level. The schematic of the CML latch is depicted in Figure 12a.

When the clock is recognized as high, the tail current flows only to the side of the differential input. Thus, the voltage of A and Ab nodes are determined by differential inputs. Conversely, when the clock is recognized as low, the tail current flows only toward the cross-coupled pair, maintaining the voltage at A and Ab nodes. In this way, it can perform the same function as latch in digital logic, because it accepts input and maintains its value. Similar to implementing a flip-flop with two latches in digital logic, CML D-FF could be made by connecting the CML latches in series and inverting only the input clock (Figure 12b). When the output of the D-FF is twisted and fed back to the input, the simplest frequency divider (FD) by 2 is implemented. Thus, the differential divider is implemented by connecting three D-FFs in series. The differential divider is realized by choosing an asynchronous scheme where the output of the previous stage goes into the clock of the next stage (Figure 12c), because the VCO frequency is so fast.

**Figure 13:**
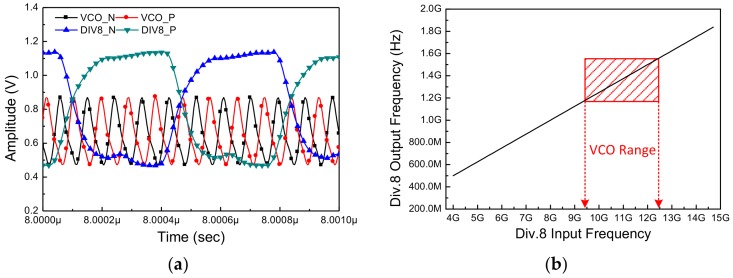
The simulation result of the differential divider (**a**) in time domain and (**b**) the range of input frequencies that can be divided by the differential divider.

Figure 13 shows the simulation results of the differential divider in the time domain. As shown in this figure, there are 8 cycles of the VCO waveform in each cycle of the divided signal. The range of input frequencies that can be divided by this block is depicted in Figure 13b. The area shown in red area indicates the output frequency range of the VCO. This ensures that the divider can cover the entire frequency range of the VCO.

**Figure 14:**
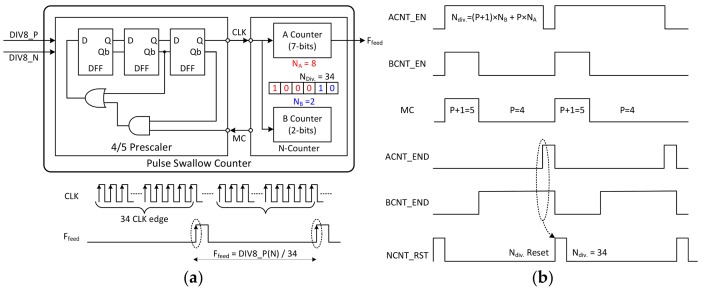
The (**a**) block diagram and (**b**) timing chart of the pulse swallow counter used in the frequency divider (FD).

The block diagram of the pulse swallow counter is described in Figure 14a, and the timing chart of this block is shown in Figure 14b. The pulse swallow counter consists of a prescaler with a P/P + 1 division ratio and an N-counter implemented with digital logic. The prescaler divides the output signals of the differential divider one more time; so the divided signal is used as the clock of the N-counter.

The N-counter performs two roles. The first role is to divide the CLK signal by the input *N_div_*. value to generate the final output of the frequency divider. The other is to determine the ratio of the prescaler by using an MC pulse. The pulse swallow counter is a commonly used for radar transceivers, because the divider might be operated at high speeds while using a low-frequency reference clock. The division ratio of the pulse swallow counter is determined by the following equation:

(11) $$N_{div.}=N_{B}\times \left(P+1\right)+\left(N_{A}-N_{B}\right)\times P$$

where *N_A_* and *N_B_* are the counting values of the A-counter and B-counter, respectively.

The prescaler is implemented with three D-FFs and simple logic functions. Like the differential divider, a CML circuit is used. The N-counter consists of a 7-bit A-counter and a 2-bit B-counter. Since the 4/5 prescaler is used, 2-bits B counter is selected. The MC signal is generated by the N-counter and fed back to the prescaler. This signal determines the division ratio of the prescaler.

As shown in the timing chart (Figure 14b), at the end of the B-counter operation, the BCNT_END signal is triggered, and the MC value changes from 1 to 0 at this moment. Accordingly, the division ratio of the prescaler is changed from 5 to 4. The ACNT_END signal is triggered when the A-counter operation has finished. When both ACNT_END and BCNT_END are high, the operation of the N-counter is initialized by the NCNT_RST signal and the same operation is repeated. The example shown in the figure is when the input of the N-counter is 34. Theoretically, a waveform of the *F_feed_* signal is generated in such a manner that a pulse is generated every 34th rising edge of CLK. The binary representation of 34, the division ratio of the N-counter, is ‘000100010′. Two bits from the least significant bit (LSB) are input to the B-counter, and the remaining bits are input to the A-counter; so the counting value of the A-counter (*N_A_*) is 8, and B-counter (*N_B_*) is 2. Substituting these values into Equation (11) yields:

(12) 34=2×5+(8−2)×4

**Figure 15:**
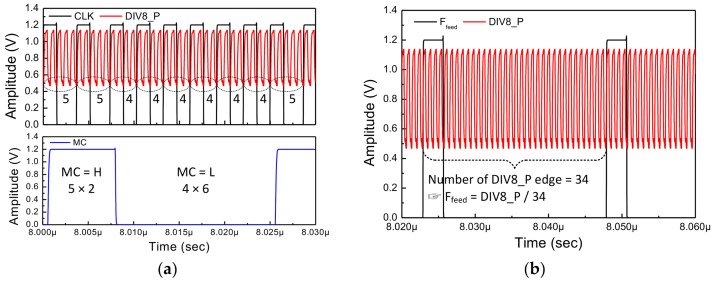
The simulation results of the pulse swallow counter in time domain. (**a**) The relationship between the Div8_P (Div8_N) signals and the CLK can be checked together with respect to changing in the MC value; (**b**) Relationship between the input and output of the pulse swallow counter.

Figure 15 shows the simulation results of the pulse swallow counter in time domain. First, the relationship between the Div8_P (Div8_N) signals and the CLK can be checked together with respect to the change in the MC value (Figure 14a). As shown in the figure, the Div_8 signal is divided into five divisions two times and four divisions six times according to the MC value. 

The graph shows the relationship between the input and output of the pulse swallow counter (Figure 15b). As a result, the relation between the frequency of the VCO and the output *F_feed_* of the FD is as follows:

(13) $$F_{feed}=F_{out}/\left(8\times N_{div.}\right)$$

#### 2.2.4. Division Ratio Controller (DRC)

The division ratio controller (DRC) consists of a sigma delta modulator (SDM) and a frequency sweep generator (FSG). Since the main function of this block is to control the division ratio of the PLL, the DRC block plays two roles in the proposed PLL. One is to express the fractional division ratio of the PLL, and the other is to control the LO in dual-mode operation. The expression of the fractional division ratio is determined by SDM, and dual-mode operation is controlled by FSG. 

**Figure 16:**
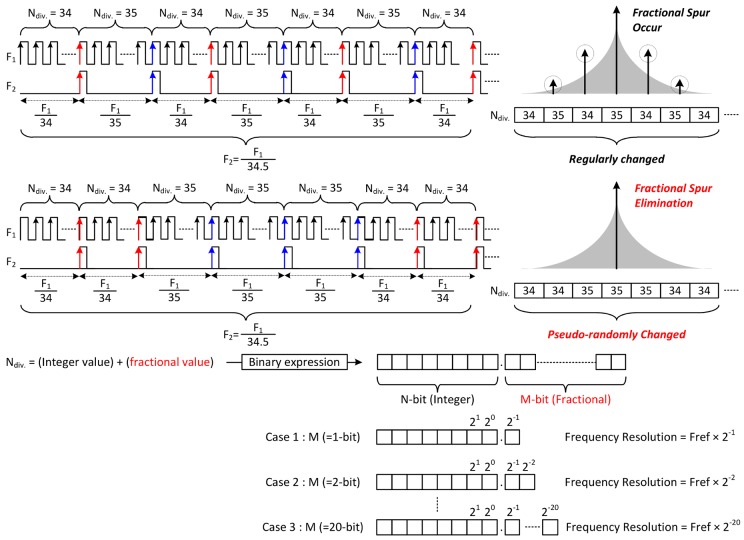
The operating principle and role of the SDM.

In digital circuits, the way to divide the frequency is to count the number of rising edges of the signal. In this way, only integer values can be selected as the division ratio. Therefore, the fractional division ratio is expressed stochastically. The example shown in the top of the Figure 16 is a method for expressing the division ratio of 34.5. As a division ratio of *F*_1_, by using 34 and 35 alternately, the frequency of *F*_2_ is substantially 34.5 divided signal of *F*_1_. The SDM represents the fractional division ratio of the PLL by combining specific division ratios. By using this method, although fractional division ratio could be expressed, another problem occurs. A specific pattern is created when 34 and 35 are selected as fixed proportions. These patterns cause fractional spurs in the frequency domain. To solve this problem, a sub-block is included in the SDM.

The below picture of the Figure 16 shows how to represent the fractional division ratio, which is another role of the SDM. The input of the SDM is the fractional part of the division ratio expressed in binary form. In Equation (9), the output frequency of the LO is determined by multiplying the reference clock by the division ratio. In other words, if the division ratio is changed by 1, the output frequency of the LO could be varied as much as the reference clock value. As can be seen from the case 1 to 3, as the number of fractional bits increases, the division ratio of the PLL can be expressed more precisely. In Equation (13), due to the differential divider used in the frequency divider (FD), the resolution of the proposed PLL is eight times larger than the resolution of the SDM. The frequency resolution of the entire PLL can is calculated as follows:

(14) $$f_{res\_PLL}=f_{res\_SDM}\times 8=\frac{38.4\times 10^{6}}{2^{20}}\times 8=292.968\;\mathrm{Hz}$$

**Figure 17:**
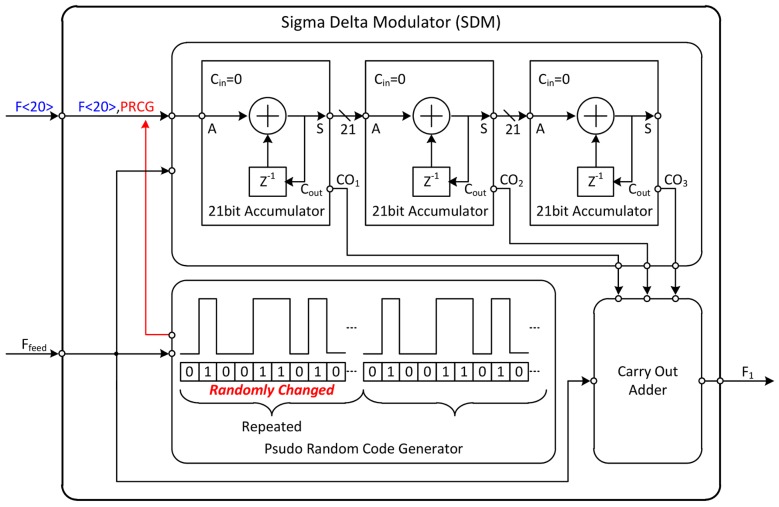
The detailed schematic of the SDM.

The detailed schematic of the SDM for the above mentioned operation is depicted in Figure 17. The fractional spur can be eliminated by using pseudo-random code generator (PRCG). This block generates a random combination of 0 s and 1 s at specific intervals. As shown in the figure, the accumulator included in the SDM has 21 bits of input. If the output of the PRCG is used as the LSB of the accumulator input, it changes the minimum value of the fractional value pseudo-randomly. Therefore, the appearance of a certain pattern can be prevented by eliminating fractional spur.

Expressing in briefly, the role of SDM is to express fractional values and to determine frequency resolution of the PLL. The ratio of the combination is extended to 20 bits for expressing division ratio more precisely. However, an error occurs, because the number of bits to be expanded is limited. Therefore, a 1-1-1 multi-stage noise shaping (MASH) type SDM structure is used [41], which removes the generated error. As shown in Figure 17, *F*_1_, which is the result of summing up the carries of each stage, is the final output of the SDM as represented by signed 4 bits. *N_div._*, which is the input of the frequency divider (FD), is the summation of the original division ratio (*N*) and the output of SDM (*F*_1_). *N_div._* is expressed as the following equation:

(15) $$N_{div.}=N+F_{1}$$

Theoretically, the range of *F*_1_ values is from −8 to 7. When the output of the PRCG is generated, the probability of successive 0 s or 1 s is much less than the probability of alternating 0 and 1. Usually *F*_1_ is determined by 0 or 1, and the remaining values are sometimes selected to eliminate the pattern.

The following is a description of how to control the dual-mode operation, which is the second function of the division ratio controller (DRC) block. As shown in Figure 6, the output (*α*) of the FSG is the additional value added to or subtracted from the dividing ratio determined by the SDM. In other words, if the FSG block is turned off, the Doppler mode is activated. Otherwise, the FMCW mode is activated. Therefore, dual-mode operation can be easily controlled with a single bit.

**Figure 18:**
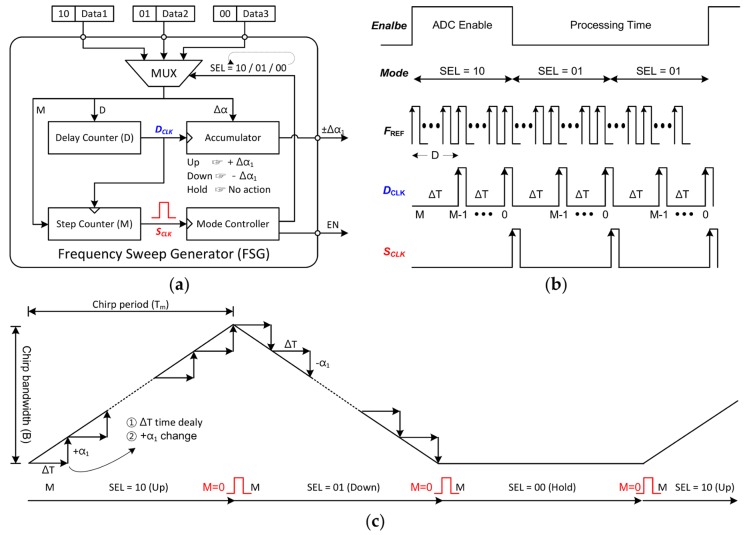
A general description of the frequency sweep generator (FSG). The detailed block diagram is shown in (**a**); The timing chart of the FSG is described in (**b**); Finally, on the time axis, the principle of how to generate FMCW waveform is expressed in (**c**).

Figure 18 provides a general explanation of the frequency sweep generator (FSG). The FSG consists of three data registers, a mode controller, an accumulator, a delay counter, and a step counter. The 2-bits shown before the data is ‘*Mode*’ mentioned in Figure 6. According to this ‘*Mode*’, a deterministic operation is performed which is stored in the data register. So this ‘*Mode*’ value is used as the selection bits of the multiplexer (MUX), and one of the data 1 to 3 must be selected by it. Once the initial operation is chosen, the next data is automatically selected by the mode controller in a predetermined order. The specified operation sequence is ‘up, down, hold’ or ‘down, up, hold’. In addition, enable (EN) signal is generated to give the information of ADC operation timing from this block to microcontroller unit (MCU). The EN signal remains high only in the first frequency chirp of the repeated sequence.

To understand more precisely operation of the FSG, a timing chart is introduced and a waveform is described on the time axis in in Figure 18b,c. The role of the delay counter is to accumulate the period of reference clock during D cycles. In other words, D means the time step until the frequency changes to the next value. Every time D_CLK_ occurs, the accumulator operates once, so the one step of frequency (∆α) changes after the time delay (∆T). The moment when the value of the step counter (*M*) becomes 0 is the moment when the single chirp ends. At this time, S_CLK_ is used as a mode-change trigger signal. Therefore, whenever the S_CLK_ is generated, the mode controller changes the selection bits of the MUX, so the next operation automatically starts. The following equations show how the chirp bandwidth (*B*) and chirp time (*T_m_*) are determined according to the input values:

(16) $$T_{m}=∆T\times M=\frac{1}{F_{ref}}\times D\times M=T_{ref}\times D\times M$$

(17) $$B=∆\alpha \times M=f_{res\_pll}\times K\times M=292.968\times K\times M$$

where the *D*, *M*, and *K* are the values stored in the data registers. These values could be set externally to produce the desired FMCW waveform. Up-, down- and hold chirp data are stored in each data 1 to data 3, respectively. The *D*, *M*, and *K* values for up-chirp are stored in data 1. Likewise, information for making down-chirp and hold time is stored in data 2 and data 3, respectively. The data consists of total of 32 bits. Two bits from the MSB are assigned to determine the operating mode and the next 16 bits represent the total step (*M*). The remaining 14 bits represent a frequency step (*K*) of 8 bits and a delay (*D*) of 6 bits. To make all this easier to understand, several examples of the input values of the FSG are summarized in the following Table 2.

**Figure 19:**
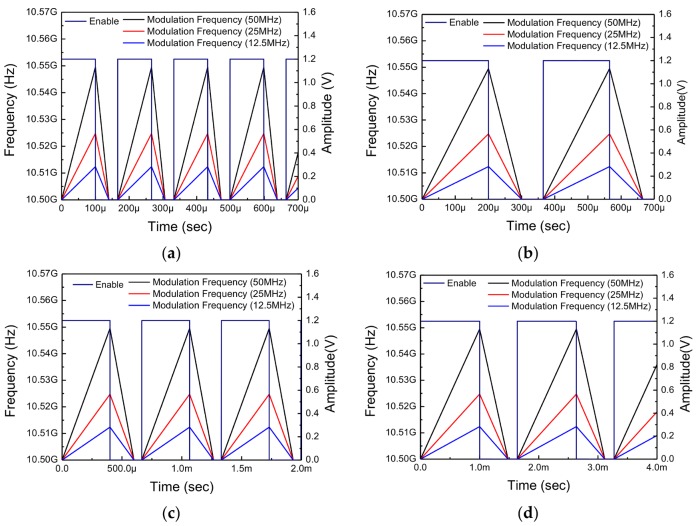
Various simulation results of FMCW waveforms. Under the same condition that chirp bandwidth is only shown at 12.5, 25 and 50 MHz, the chirp time is varied in (**a**) 0.1 ms; (**b**) 0.2 ms; (**c**) 0.4 ms; and (**d**) 1 ms.

The simulation results of FMCW waveform under various conditions are shown in Figure 19. Under the same condition in which chirp bandwidth is only 12.5, 25, and 50 MHz, the chirp time is varied in 0.1 ms, 0.2 ms, 0.4 ms, and 1 ms.

## 3. Results

**Figure 20:**
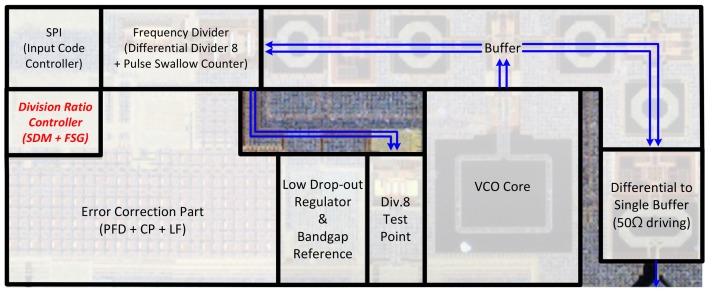
Photograph of the proposed dual-mode LO.

The proposed dual-mode LO for motion detecting sensor is implemented using a 1P8M 0.13 μm TSMC CMOS process. Figure 20 shows a photograph of the fabricated dual-mode LO. The chip size is 1.75 × 0.6 mm^2^ excluding all the pads.

The output frequency and the phase noise plot are obtained by an Agilent E4440A spectrum analyzer. Also, the output frequency chirp signals in time domain are collected by Keysight E5052B signal source analyzer.

**Figure 21:**
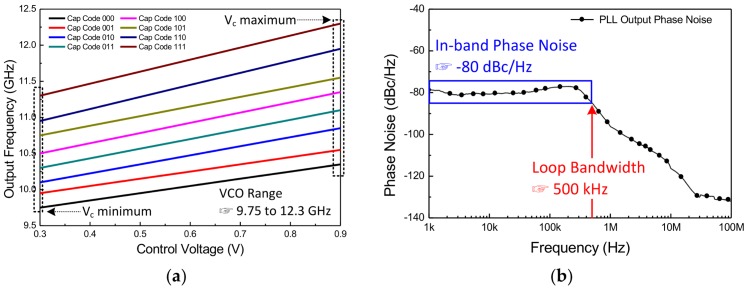
The output frequency of the VCO is shown in (**a**); and the measured phase noise performance at the frequency of 10.525 GHz is described in (**b**).

Under the 1.2-V supply voltage, the output frequencies of VCO are described as a function of the varactor control voltage and the capacitor bank code (Figure 21a). The measured frequency range is 9.75–12.3 GHz with the tuning range of 2.55 GHz (23.13%). The frequency range is slightly different between the simulation and the measurement result because, when the VCO is used with PLL, the range of the control voltage is limited by the charge pump. Also, the gain of the VCO gradually increases as the capacitor bank code becomes higher, since the influence by the parasitic capacitance is reduced. Finally, there is an overlap interval between the capacitor codes to generate all frequencies within the variable range without error when AFC operates. The measured phase noise plot at the fixed frequency of 10.525 GHz condition. The loop bandwidth of the proposed architecture is 500 kHz and the in-band phase noise performance is about −80 dBc/Hz at 10 kHz offset frequency, as shown in Figure 21b.

**Figure 22:**
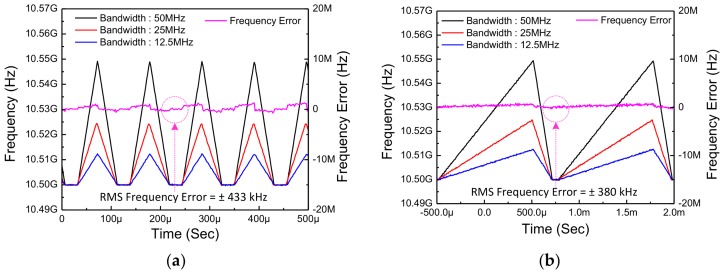
The measured FMCW chirp waveform and root-mean square (FMS) frequency error in time domain according to several conditions with a chirp time of (**a**) 100 μs and (**b**) 1 ms, according to modulation bandwidth of 12.5 MHz, 25 MHz, and 50 MHz.

Figure 22 shows the measured FMCW chirp waveform and root-mean square (RMS) frequency error in time domain in the FMCW mode according to several conditions. As a representative example, the output waveform with a chirp time of 100 μs and modulation bandwidth of 12.5 MHz, 25 MHz, and 50 MHz is described in Figure 22a. Under the same modulation bandwidth, an output waveform with a chirp time of 1ms is depicted in Figure 22b. The RMS frequency errors are ±433 kHz and ±380 kHz, respectively. Since the amount of frequency change per step in slow-chirp condition (1 ms) is much smaller than fast-chirp condition (100 μs), the RMS frequency error is a smaller in the slow-chirp condition.

**Figure 23:**
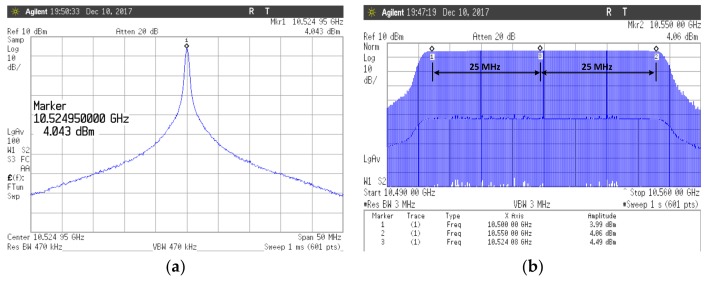
The output spectrum of (**a**) the Doppler mode in which the output frequency is fixed and (**b**) the FMCW mode with the 50 MHz modulated bandwidth.

**Figure 24:**
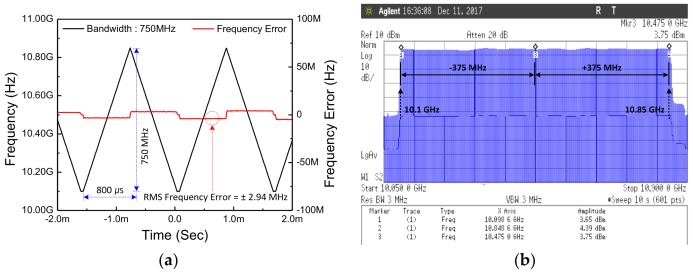
The waveform of frequency chirp with 750 MHz bandwidth is shown in (**a**) time domain and (**b**) frequency domain.

Figure 23a shows the spectrum of the Doppler mode in which the output frequency is fixed as a result measured in the frequency domain. The output power at the frequency of 10.525 GHz is 4 dBm. The output spectrum of the FMCW mode with the 50 MHz frequency modulation is shown in Figure 23b. The Figure 24 shows the measurement result of the maximum modulated bandwidth (750 MHz) in time and frequency domain when the proposed LO is operated in FMCW mode (Figure 24a,b).

Comparison of the results for the proposed structure and previously reported studies are summarized in Table 3. Compared with [11], the proposed architecture seems to have a large chip size and power consumption. However, the proposed structure includes a buffer to drive the antenna and the front-end mixer. Also considering the difference in process and the amount of power consumed in the core, the proposed structure has sufficiently good performance.

## 4. Conclusions

In this paper, a dual-mode LO is proposed in which a CW radar could be operated in either Doppler or FMCW mode. This structure is proposed to solve the problems that might occur when the CW radar operates in one mode only. The main features are that the FMCW radar is implemented by division ratio control method, and dual-mode operation is possible with only 1-bit control. The advantage of this structure is that the radar sensor can be implemented more simply, with less power consumption than in the structure using DDS. As shown in the comparison table, the proposed architecture has comparable performance in various categories such as power consumption, chip size, modulation bandwidth and frequency tuning range. In addition, the proposed LO is valuable as a way to implement a fully-integrated RF transceiver, because all the components are all built into the chip. Therefore, the proposed dual-mode LO is suitable for small radar sensors that can provide various functions for motion detection such as, automatic lighting control system, and unmanned security system.

## Figures and Tables

**Table 1:** Parameters of the proposed dual-mode LO.

Specification	Value
Output frequency (GHz)	10.525 (Doppler mode)
	10.5 to 10.55 (FMCW mode for X-band)
	10.1 to 10.85 (Maximum modulation range)
	9.7–12.4 (Entire LO output range)
Reference clock (MHz)	38.4
PLL loop bandwidth (kHz)	500
PLL in-band phase noise (dBc/Hz)	−80
Chirp time (ms)	0.1 to 1

**Table 2:** Parameters of the FSG block for FMCW operation.

*T_REF_* (ns)	*F_pll_res_* (Hz)	*M*	*D*	*K*	Δ*α* (kHz)	Δ*T* (ns)	*B* (MHz)	*T_m_* (μs)
26	293	768	5	222	65.039	130	50	100
		768	5	111	32.519	130	25	100
		768	5	55	16.113	130	12.5	100
		768	10	222	65.039	260	50	200
		768	10	111	32.519	260	25	200
		1536	10	111	32.519	260	50	400
		1536	25	111	32.519	651	50	1000

**Table 3:** Comparison result of previously reported studies.

Reference	[1]	[10]	[11]	[28]	This	Unit
Technology	130	180	65	130	130	nm
Process	SiGe	CMOS	CMOS	CMOS	CMOS	-
Method	DDS-based	DDS-based	Div. ^1)^	Div. ^1)^	Div. ^1)^	-
Frequency	8.65–8.665	10.5–10.55	8.4–9.4	8.2–8.25	9.7–12.3	GHz
Mod. Bandwidth ^2)^	12.5–50	50	940	50	0–750	MHz
Chirp time	-	-	0.05–0.22	0.1	0.1–1	ms
Max. RMS freq. error	-	-	±1.9	-	±2.94	MHz
PLL loop bandwidth	0.1	-	5	0.5	0.5	MHz
Phase noise (@10 kHz)	−86.45	-	−105	−76	−80	dBc/Hz
Phase noise (@1 MHz)	−114.04	−93	−105	−98.85	−96.25	dBc/Hz
Power consumption	333 ^3)^/326 ^4)^	350	14.8	-	18 ^5)^/114 ^6)^	mW
Area	8.75	8.58	0.18	5.04	1.05	mm^2^

^1)^ Div. expresses division ratio control method; ^2)^ The allocated bandwidth is 50 MHz in X-band radar application; ^3)^ Power consumption is expressed when operated in transmitting mode and ^4)^ receiving mode; ^5)^ Power consumption is expressed when considered core circuit only and ^6)^ including 50 Ω load buffer.
